# Association of Self-DNA Mediated TLR9-Related Gene, DNA Methyltransferase, and Cytokeratin Protein Expression Alterations in HT29-Cells to DNA Fragment Length and Methylation Status

**DOI:** 10.1155/2013/293296

**Published:** 2013-12-29

**Authors:** István Fűri, Ferenc Sipos, Sándor Spisák, Gergő Kiszner, Barnabás Wichmann, Andrea Schöller, Zsolt Tulassay, Györgyi Műzes, Béla Molnár

**Affiliations:** ^1^2nd Department of Internal Medicine, Semmelweis University, Szentkirályi Street 46, Budapest 1088, Hungary; ^2^Molecular Medicine Research Unit, Hungarian Academy of Sciences, Nádor Street 7, Budapest 1051, Hungary; ^3^1st Department of Pathology and Experimental Oncology, Semmelweis University, Üllői Street 26, Budapest 1085, Hungary

## Abstract

To understand the biologic role of self-DNA bound to Toll-like Receptor 9 (TLR9), we assayed its effect on gene and methyltransferase expressions and cell differentiation in HT29 cells. HT29 cells were incubated separately with type-1 (normally methylated/nonfragmented), type-2 (normally methylated/fragmented), type-3 (hypermethylated/nonfragmented), or type-4 (hypermethylated/fragmented) self-DNAs. Expression levels of TLR9-signaling and proinflammatory cytokine-related genes were assayed by qRT-PCR. Methyltransferase activity and cell differentiation were examined by using DNA methyltransferase (DNMT1, -3A, -3B) and cytokeratin (CK) antibodies. Treatment with type-1 DNA resulted in significant increase in TLR9 expression. Type-2 treatment resulted in the overexpression of TLR9-related signaling molecules (MYD88A, TRAF6) and the IL8 gene. In the case of type-3 treatment, significant overexpression of NFkB, IRAK2, and IL8 as well as downregulation of TRAF6 was detected. Using type-4 DNA, TRAF6 and MYD88A gene expression was upregulated, while MYD88B, IRAK2, IL8, and TNFSF10 were all underexpressed. CK expression was significantly higher only after type-1 DNA treatment. DNMT3A expression could also be induced by type-1 DNA treatment. DNA structure may play a significant role in activation of the TLR9-dependent and even independent proinflammatory pathways. There may be a molecular link between TLR9 signaling and DNMT3A. The mode of self-DNA treatment may influence HT29 cell differentiation.

## 1. Introduction

Toll-like receptors (TLRs) belong to a family of innate immune receptors that detect and clear invading microbial pathogens. Intracellular TLRs (TLR3, TLR7, TLR8, and TLR9) specifically recognize nucleic acids such as double-stranded ribonucleic acid (RNA), single-stranded RNA, and CpG deoxyribonucleic acid (DNA) derived from microbial components [1]. This identification serves as a very important tool linking innate and adaptive immune responses [2].

TLR9 expressing cells in the colonic mucosa such as monocytes, macrophages, dendritic cells, and some types of B cells [3, 4] usually accumulate within the area of isolated lymphoid follicles and lymphoid aggregates [5]. However, some TLRs (i.e., TLR4, -5, and -9) may also be expressed by modified epithelial cells [6]. HT29 colon carcinoma cells also express TLR9 that can be activated by pathogen-originated DNA sequences [7]. Apical epithelial TLR9 activation by bacterial DNA fragments has been reported to maintain colonic homeostasis [8]. TLR9 could be also activated by both self-DNA and synthetic oligodeoxynucleotides, which contain unmethylated CpG sequences [9–11]. The activation of TLR9-signaling eventually results in pro- and anti-inflammatory cytokine production and enhanced B-cell proliferation [12]. Though ligation of TLR9 is likely to stimulate widespread protective innate immune responses, the spectrum of TLR9-dependent gene expression is not understood. CpG DNA binding to TLR9 is known to enhance innate immunity. However the magnitude, duration, and scope of CpG-DNA-induced changes concerning gene expression are not clarified, despite the extensive studies of TLR9-mediated signaling networks. It has been found that a single-dose CpG DNA injection *in vivo *can trigger an initial rapid rise in gene activation, like in mRNA levels, which return to a basic level within 2-3 days [13], but the immune reactivity will be further affected for several weeks [14, 15]. Moreover, it has already been observed that, after introduction of CpG DNA, the initial gene activation displays two induced peaks: after 3 hours and after 5 days, respectively [16]. In the case of MDA-MB-231 human breast cancer cell line, sequence modifications to the CpG oligonucleotides that targeted the secondary structures were shown to influence the invasion-inducing effect. In contrast, methylation of the cytosine residues of the parent CpG oligonucleotide did not affect the TLR9-mediated invasion compared with the unmethylated parent CpG oligonucleotide [17].

Abnormal methylation patterns are associated with certain human tumors and developmental abnormalities. In the pathogenesis of colorectal neoplasias, three major DNA methyltransferases (DNMT) have been proposed to be involved: DNMT1, -3A, and -3B [18]. DNMT1 is responsible for maintenance of the DNA methylation pattern after DNA replication. The DNMT3 family (3A, 3B, and 3L) primarily acts on *de novo* methylation during gametogenesis and development. Moreover, it can serve cooperatively with DNMT1 to regulate DNA methylation maintenance. Human colon cancer cell lines (including HT29) are hypermethylated on the distal DNMT3B promoter as compared to healthy colon tissues, correlating with the low expression level that results in hypomethylation of many of its target gene promoters [18], which has importance in the etiology of sporadic CRC.

Currently, no concrete data exists on how the characteristics of TLR9 activating self-DNA fragments (i.e., fragment length, methylation status) influence the downstream signaling pathways, cytokine response, cell differentiation, and expression of DNMTs which may have important immunobiologic consequences in the case of inflammatory and tumorous colonic disorders.

In this study, we assayed the initial, short-term TLR9-associated gene expression effects of purified, differently fragmented, and methylated self-DNA sequences on HT29 colon carninoma cells. We also examined the association between the induction of TLR9 signaling and the expressions of DNA methyltransferases and cytokeratin (CK) after self-DNA treatment.

## 2. Materials and Methods

### 2.1. HT29 Cell Culture

HT29 colon adenocarcinoma cells were purchased from the 1st Department of Pathology and Experimental Oncology, Semmelweis University, Budapest, Hungary. Cells were cultured in a specific pathogen-free cell culture laboratory.

HT29 cells were maintained in RPMI 1640 (Sigma-Aldrich, Saint Louis, USA) and supplemented with 10% (v/v) fetal bovine serum (FBS; Standard Quality; PAA Laboratories GmbH, Pasching, Austria), 160 *μ*g/mL gentamycin (Sandoz, Sandoz GmbH, Austria), and 125 *μ*g/mL amphotericin B (Sigma). Media were replaced every second day.

### 2.2. Isolation, Methylation, and Fragmentation of Genomic DNA

Genomic DNA was isolated from 5 × 10^7^ HT29 cells with High Pure PCR template preparation kit containing proteinase K (Roche GmbH, Germany). Then the DNA samples were treated with 5 *μ*L RNase A/T1 Mix (Thermo Scientific, Germany). DNA concentration was determined by Nanodrop (Thermo Scientific, Germany).

Genomic DNA was divided into four equal shares.

1. The first share was neither artificially hypermethylated nor fragmented (type 1. DNA; nMnF).
2. The second share was fragmented by ultrasonic fragmentation for 2 minutes (type 2. DNA; nMF).
3. The third share was artificially hypermethylated (type 3. DNA; MnF) using CpG methyltransferase M.SssI (New England Biolabs Ipswich, USA). M.SssI methylates all cytosine residues (C5) within the double-stranded dinucleotide recognition sequence 5′…CG…3′ [19].
4. The fourth share was both artificially hypermethylated and fragmented (type 4. DNA; MF).

**Figure 1:**
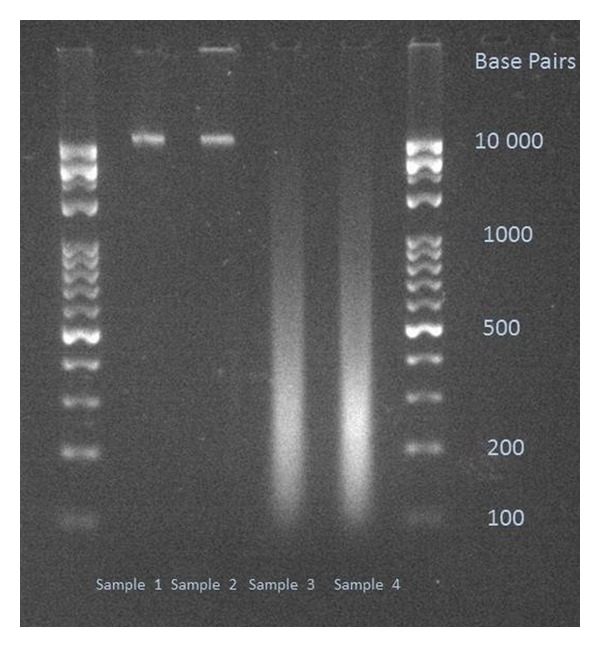
Electrophoretic gel image of the used DNA samples. Sample 1: nMnF DNA; Sample 2: MnF DNA; Sample 3: nMF DNA; Sample 4: MF DNA. 2% Tris-Borate-EDTA (TBE) gel.

The length of the fragmented DNA shares was determined by agarose gel electrophoresis (Figure 1).

### 2.3. DNA Treatment and Isolation of Total RNA

0.5 × 10^6^ HT29 cells were placed into a six-well treatment plate in RPMI 1640 and supplemented with gentamycin, amphotericin B, and FBS, as described earlier. After 24 hours, the starting medium was changed to RPMI 1640, with gentamycin and without FBS. Then 15 *μ*g of each of the different types of pretreated DNA was separately dissolved in 200 *μ*L of sterile phosphate buffered saline (PBS). The cells were then treated with the dissolved DNA samples which were free of protein, RNA, or lipopolysaccharide contamination. For nontreated controls, only 200 *μ*L of sterile PBS was added. Cells were incubated at 37°C in a 5% CO_2_ atmosphere and 95% humidity. After 24 hours, cells were washed twice in 5 mL sterile PBS. After the second washing, cells were resuspended in 5 mL PBS as a final volume. 2.5 mL of cell suspension was used for total RNA isolation, and the rest of the cell suspension was used for immunocytochemistry.

Total RNA from the isolated HT29 cells was extracted with the RNeasy Mini Kit (Qiagen, USA) according to the prescription of the manufacturer.

### 2.4. Reverse Transcription and Quantitative Real-Time Polymerase Chain Reaction

Genes of TLR9-associated signaling and interleukin-8 (IL8) as an indicator of epithelial TLR9-pathway induction were selected, and then oligonucleotide primers were designed. After quantitative (Nanodrop) and qualitative analysis (Bioanalyzer Pico 600 chip kit RNA program; RIN > 8 in all cases), reverse transcription was performed using 1 *μ*g of total RNA (High Capacity cDNA Reverse Transcription Kit, Applied Biosystems, USA). Quantitative real-time (qRT) PCR was performed using Probes Master and SYBR green (Roche GmbH, Germany). Gene expression levels for each individual sample were normalized to 18S expression. Mean relative gene expression was determined and differences were calculated using the 2-ΔC(t) method. The list of oligonucleotide primers used is detailed in Table 1.

### 2.5. Immunocytochemistries

From every cell suspension, 2.5 mL was used for immunocytochemical (ICC) analyses. Cells were centrifuged on a glass slide, fixed in acetone at −20°C for five minutes, and then incubated in 1% bovine serum albumin/10% normal goat serum/0.3 M glycine in 0.1% PBS-Tween for one hour to permeate the cells and block nonspecific protein-protein interactions.

#### 2.5.1. TLR9 ICC

Cells were incubated overnight at 4°C with mouse anti-human monoclonal anti-TLR9 antibody (LS-B2341, clone: 26C593.2; working dilution: 20 *μ*g/mL; LifeSpan BioSciences, USA). Human lung tissue was used as positive control.

#### 2.5.2. DNMT1 ICC

Cells were incubated overnight at 4°C with rabbit monoclonal anti-DNMT1 antibody (Cat. No.: 2788-1, clone: EPR3522; working dilution 1 : 50; Epitomics-Abcam, USA). HeLa cells were used as positive controls.

#### 2.5.3. DNMT3A ICC

Cells were incubated overnight at 4°C with monoclonal anti-DNMT3a antibody (64B1446, working dilution: 2 *μ*g/mL; Abcam, USA). HeLa cells were used as positive controls.

#### 2.5.4. DNMT3B ICC

Cells were incubated overnight at 4°C with rabbit polyclonal anti-DNMT3B antibody (NBP1-00783, working dilution: 1 : 100; Novus Biologicals, UK). Human hepatocellular carcinoma tissue was as positive control.

#### 2.5.5. Pan-Cytokeratin ICC

Cells were incubated for one hour at 37°C with mouse monoclonal anti-pan cytokeratin antibody (ab6401, clone: PCK-26, working dilution: 1 : 200, Abcam, USA). Human healthy colonic mucosa was used as positive control.

A Biotin-conjugated Goat polyclonal (1/1000) was used as the secondary antibody. Signal conversion was carried out with the Liquid DAB + Substrate Chromogen System (DAKO). After the final rinsing in PBS, hematoxylin costaining was performed.

#### 2.5.6. ICC Evaluation

Slides were digitalized using a high-resolution Mirax Desk instrument (Zeiss, Goettingen, Germany) and analyzed with the Mirax TMA Module software (Zeiss). 1000 cells/slides were evaluated. In the case of TLR9 and CK, cytoplasmic immunoreactions were scored as negative (−), mild (+), moderate (++), and strong (+++). DNMT nuclear/cytoplasmic expressions were scored as negative (−), mild (+), moderate (++), and strong (+++) immunoreactions. The number of immunoreactive cells was counted.

### 2.6. Statistical Analysis

The data was expressed as the mean ± SD. Chi^2^-test and Student's *t*-test were used for statistical analyses. *P* < 0.05 was considered statistically significant.

## 3. Results

### 3.1. Effect of DNA Treatment on Gene Expression

Although all of the assayed genes showed significant gene expression alteration following DNA treatments, the characteristics of the expression profiles altered in every case. Based on the type (i.e., hypermethylated and/or fragmented) of DNA used, different genes were over- or underexpressed in the treated cells.

After incubation with nMnF (type 1) DNA, mTLR9 was significantly overexpressed (*P* = 0.0298). In contrast, MYD88A (*P* = 0.006) and TRAF6 (*P* = 0.0042) genes were downregulated.

In the case of nMF (type 2) DNA treatment, TLR9 (*P* = 0.0072), MYD88A (*P* = 0.0304), TRAF6 (*P* = 0.0016), and IL8 (*P* = 0.0087) genes were overexpressed, while MYD88B (*P* = 0.012) and IRF7B (*P* = 0.002) were downregulated.

After MnF (type 3) DNA treatment, NF*κ*B (*P* = 0.001), IRAK2 (*P* = 0.024), and IL8 (*P* = 0.0002) genes were overexpressed. However, TRAF6 showed downregulation (*P* = 0.004).

Incubation with MF (type 4) DNA resulted in overexpression of TRAF6 (*P* = 0.006) and MYD88A (*P* = 0.0033) and downregulation of MYD88B (*P* = 0.0023), IRAK2 (*P* = 0.004), TNFSF10 (*P* = 0.0034), and IL8 (*P* = 0031) genes.

**Figure 2:**
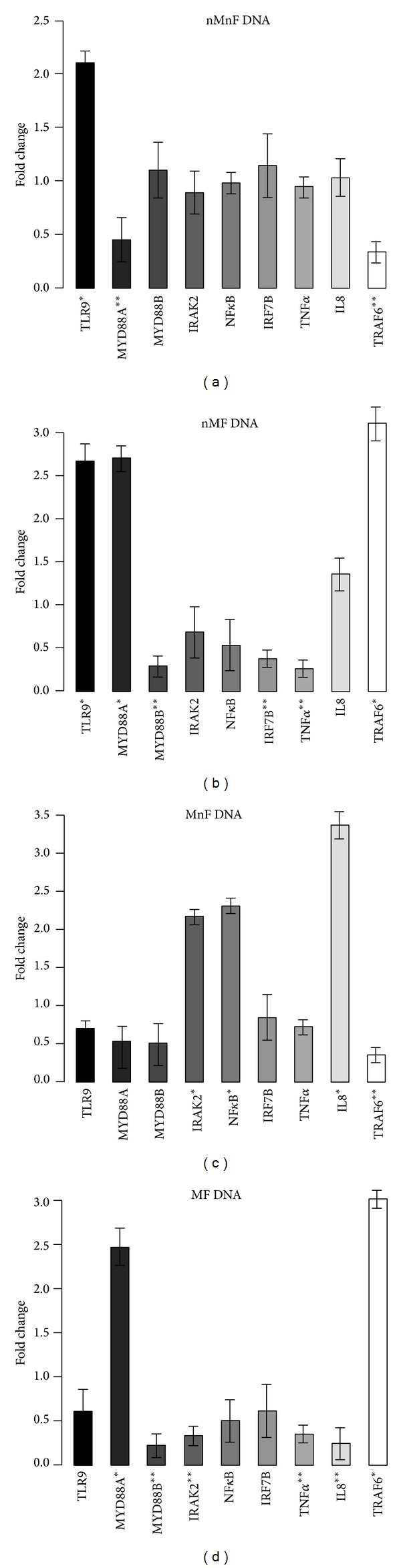
Expression fold changes of the assayed genes. (a) Incubation with normally methylated/nonfragmented DNA. TLR9 was overexpressed, and MYD88A and TRAF6 were downregulated. (b) Incubation with normally methylated/fragmented DNA. TLR9, MYD88A, and TRAF6 were overexpressed, and MYD88B, IRF7B, and TNF-alpha were downregulated. (c) Incubation with hypermethylated/nonfragmented DNA. NF*κ*B, IRAK2, and IL8 were overexpressed and TRAF6 was downregulated. (d) Incubation with hypermethylated/fragmented DNA. MYD88A and TRAF6 were overexpressed, and MYD88B, IRAK2, and IL8 and TNF-alpha showed downregulation. 18S was used as a housekeeping gene. Genes with >2 expression fold changes are marked with *; the ones with <0.5-fold change are marked with **.

It is notable that only incubations with hypermethylated DNAs (types 3 and 4) resulted in significant expression changes of the IL8 gene, the indicator of epithelial TLR9-pathway induction. Gene expression alterations are visualized in Figure 2.

### 3.2. Effect of DNA Treatment on Protein Expressions

At protein level, statistically significant expression alterations were only found after incubation with MnF (type 3) DNA.

**Figure 3:**
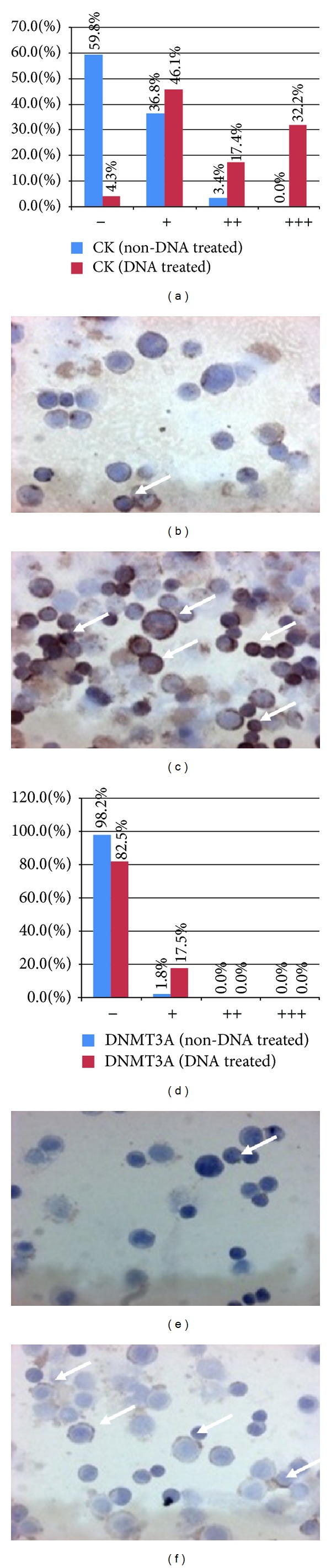
Distribution of cytokeratine (CK; (a)–(c)) and DNA methyltransferase 3A (DNMT3A; (d)–(f)) immunoreactive HT29 cells. (a) and (d) *x*-axes represent the intensities of immunoreactions from negative (−) to strong (+++). *y*-axes represent the distribution (percentage) of cells showing different immunoreaction intensities (blue columns: control cells; red columns: type 3. DNA-treated cells). (b) and (c) CK expression before (b) and after (c) type 3. DNA incubation (immunoreactive cells/arrows/display brownish cytoplasmic reaction, 200x magnification, hematoxylin costaining). (e) and (f) DNMT3A expression before (e) and after (f) type 3. DNA incubation (immunoreactive cells/arrows/displayed brownish cytoplasmic reaction, 200x magnification, hematoxylin costaining).

After MnF DNA treatment, CK immunoreaction was significantly increased in treated cells compared to the control ones (*P* < 0.0001; Figures 3(a)–3(c)).

The expression of DNMT3A was induced by MnF DNA treatment compared to untreated controls (*P* < 0.0001; Figures 3(d)–3(f)).

No changes in DNMT1 and DNMT3B immunoreactions were observed after type 1, 2, and 4 DNA incubations.

**Figure 4:**
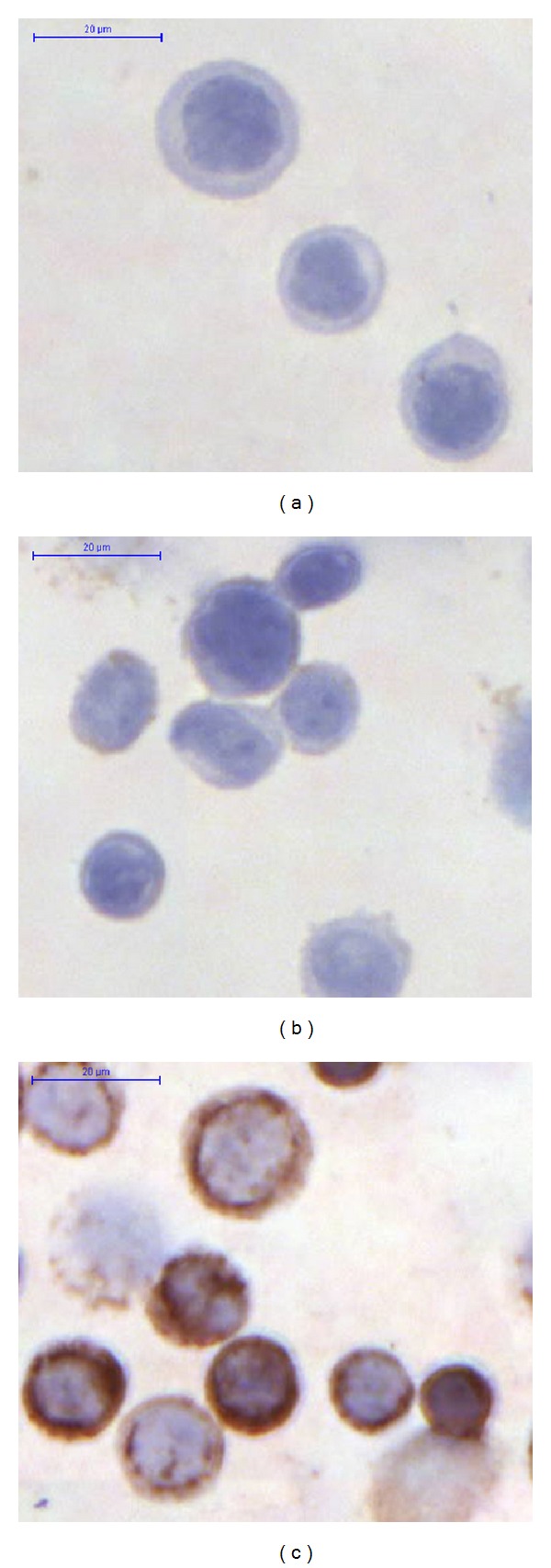
Toll-like receptor 9 immunocytochemistry. (a) Negative control; (b) immunonegative HT29 cells; (c) immunopositive (brownish immunoreaction) HT29 cells (200x magnification, hematoxylin costaining).

Similar to mRNA expressions, the number of TLR9 immunoreactive HT29 cells was significantly elevated after type 1 (48.74% ± 6.7%) and type 2 (39.24% ± 4.86%) DNA treatments compared to nontreated controls (8.96% ± 2.48%; *P* < 0.001). TLR9 cellular immunoreaction can be seen in Figure 4.

## 4. Discussion

Toll-like receptor 9 is a mediator of innate immunity that is capable of detecting DNA from both endogenous and microbial sources [1]. Beyond immune cells, recent data suggests that TLR9 expression can also be detected in various normal and tumorous cells, including HT29 colon carcinoma cells [7, 20–24]. It has been recently shown that TLR9 stimulation with agonistic CpG sequences stimulates invasion in various cancer cells [20, 24] via MYD88-independent and TRAF6 partially dependent pathways [17].

The aim of this study was to investigate how the characteristics of self-DNA influence TLR9 signaling, DNA-methyltransferase expression, and differentiation of HT29 cells. By using M.SssI enzyme, the normal physiologic methylation pattern of self-DNA was artificially modified, namely, hypermethylated.

Our results indicate that incubation of HT29 colon carcinoma cells with differently pretreated self-DNA results in different types of gene expression and cellular responses. While normally methylated nonfragmented (so called “genomic”) self-DNA mainly activated the MYD88-dependent TLR9 signaling pathway, hypermethylation of self-DNA activated IL8 production as well. Hypermethylation of self-DNA shifted MYD88-dependent signaling to the MYD88-independent pathway with primarily an inhibitory effect, while hypermethylation and fragmentation of self-DNA had mainly an inhibitory effect on both the key elements of MYD88-dependent TLR9 signaling and IL8 expression. The overexpression of MYD88A and TRAF6 after type 4 DNA treatment may indicate the involvement of other, currently not assayed Toll-like receptor-signaling pathways.

Originally, TLR9 was characterized as a receptor that detects unmethylated CpG sequences within microbial DNA [25]. It is known that epithelial TLR9 could be activated by synthetic oligodeoxynucleotides [8–11]. Therefore, in this study, modifications to self-DNA in both length and methylation status were used to probe structural features and their correlation with TLR9 activation. Our results suggest that DNA structure (e.g., methylation status and fragment length) plays a significant role in activation of the TLR9-mediated signaling pathways. Similar immunobiologic effect of DNA structure on cell invasion assays has been already described [13]. Further biophysical studies are needed to characterize the structural characteristics of oligonucleotides that are needed to induce a TLR9-mediated inflammatory response.

Depending on the type of self-DNA, the protein expression of DNA methyltransferase 3A was also altered. Our finding indicates that there may be a molecular link between TLR9 signaling and DNMT3A. Emerging studies found that the levels of DNMTs' mRNA are increased in various malignancies, including colorectal, hepatocellular, and gastric cancer [26–28]. It was previously shown that gastrointestinal cancers are characterised by high levels of DNMTs and a low demethyltransferase expression [29]. DNMTs and demethyltransferase cooperate with each other, leading to genetic instability [30] that eventually promotes cancer progression [31]. In lung cancer, it was recently shown that the overexpression of DNMT3A protein was significantly associated with a lower overall survival [32].

In cancer cells, the DNA methylation pattern becomes altered, resulting in a cluster of genes to undergo promoter hypermethylation and become transcriptionally silent. Reexpression of methylation silenced genes by influencing DNA methyltransferase expression and function has emerged as an effective therapeutic strategy in different disorders [33]. One cannot exclude that in the case of physiological/pathological circumstances, self-DNA released by necrotic/apoptotic cells may have paracrine effects on neighbour cells through TLR9 signaling, and the methylation status of the bound DNA is reflected in the cell's response. It is probable that the released self-DNA is a biologically active molecule in a local context.

Our study also demonstrates that the mode of self-DNA treatment influences the differentiation of HT29 cells. Cytokeratin expression is usually very low in HT29 colon carcinoma cells as this cell line is nondifferentiated. The fact that self-DNA treatment promotes cytokeratine expression of HT29 cells has very important clinical merit. Using self-DNA to differentiate aggressive carcinomas into less aggressive cancers appears to be a promising novel approach to the treatment of colorectal cancer and could represent an important alternative therapeutic choice in the future.

The fact that only incubations with hypermethylated DNAs (types 3 and 4) resulted in significant expression changes of the IL8 gene, the indicator of epithelial TLR9-pathway induction, and only treatment with type-3 (MnF) DNA resulted in CK and DNMT protein expression changes indicates that the methylation status of self-DNA may represent a more prominent factor than fragment length regarding its biologic effect. However, as there can be considerable differences between (colon) cancer cell lines (e.g., tumor growth and metastatic ability), our results have to be confirmed in other cell lines as well.

In conclusion, our study suggests that the biologic effect of HT29 colon carcinoma cell line-derived self-DNA on TLR9-relating signaling, methyltransferase expression, and cell differentiation depends on the fragment length and methylation status of DNA; notwithstanding the methylation pattern seems to display a more remarkable biologic effect. Further studies are required to establish the exact structural and biophysical properties of the inflammation-inducing DNA sequences and the roles that these pathways play in the pathophysiology of colonic disease.

## Figures and Tables

**Table 1:** List of the oligonucleotide primers used.

Toll-like receptor 9-related signaling (MYD88-dependent pathway)	
Toll-like receptor 9	F: CAATGTCACCAGCCTTTCCT R: CAGGTGGGCAAAGTCAGAAT

MYD88A	F: GAAGAAAGAGTTCCCCAGCA R: GTGCAGGGGTTGGTGTAGTC

MYD88B	F: CTCCTCCACATCCTCCCTTC R: CGCACGTTCAAGAACAGAGA

IRAK2	F: CTTGGAGTGGGCTTTCTGAG R: AGGCTGGAATTGTCAACGTC

TRAF6	F: CTTTGGCAAATGTCATCTGTG R: CTGAATGTGCATGGAATTGG

NF*κ*B	F: TATGTGGGACCAGCAAAGGT R: GCAGATCCCATCCTCACAGT

TNFSF10	F: ATCTGGGACGGTGCTGTAAC R: CAGGGCAGACATACACTGTCA

Toll-like receptor 9-related signaling (MYD88-independent pathway)	

IRF7B	F: GGGTGTGTCTTCCCTGGATA R: GCTCCATAAGGAAGCACTCG

Epithelial cell-derived proinflammatory chemokine (neutrophil chemotactic factor)	

IL8	F: GTGCAGTTTTGCCAAGGAGT R: AAATTTGGGGTGGAAAGGTT
